# *MMP13* mRNA Expression Level as a Potential Marker for Knee OA Progression—An Observational Study

**DOI:** 10.3390/jcm14041263

**Published:** 2025-02-14

**Authors:** Kamila Baran, Aleksandra Czechowska, Karolina Kopacz, Gianluca Padula, Monika Migdalska-Sęk, Wiesław Tomaszewski, Krzysztof Nowak, Marcin Domżalski, Ewa Brzeziańska-Lasota

**Affiliations:** 1 Department of Biomedicine and Genetics, Biology and Medical Microbiology, Medical University of Lodz, 92-215 Lodz, Poland; monika.migdalska-sek@umed.lodz.pl (M.M.-S.); ewa.brzezianska@umed.lodz.pl (E.B.-L.); 2 Academic Laboratory of Movement and Human Physical Performance, Medical University of Lodz, 90-001 Lodz, Poland; aleksandra.czechowska@umed.lodz.pl (A.C.); karolina.kopacz@umed.lodz.pl (K.K.); gianluca.padula@umed.lodz.pl (G.P.); 3 Foundation for Medical Education, Health Promotion, Art and Culture ARS MEDICA, 03-721 Warsaw, Poland; w.tomaszewski@wp.pl; 4 Department of Orthopedics and Traumatology, University Clinical Hospital No. 2 of the Medical University of Lodz, 90-549 Lodz, Poland; krzysztof.nowak@umed.lodz.pl (K.N.); marcin.domzalski@umed.lodz.pl (M.D.)

**Keywords:** osteoarthritis, *MMP13*, visual analogue scale, real-time polymerase chain reaction

## Abstract

**Background/Objectives:** Osteoarthritis (OA) is a very common degenerative joint disease that has a significant negative impact on patients’ lives and which can lead to functional limitations and disability. Matrix metalloproteinase 13 (MMP-13) is a key enzyme responsible for the degenerative changes in cartilage occurring during the pathogenesis of OA. This cohort study analyzed the differences in the expression level of *MMP13* mRNA in articular cartilage with subchondral bone and in the synovium of patients with OA, according to the disease stage, in order to develop potential markers for OA progression, as well as for the degree of pain perception, in order to discover a molecular biomarker related to pain. **Methods:** In thirty-one patients (n = 31), the expression level of the studied gene was assessed in the affected and unaffected areas of the knee joint using the qPCR method. Statistical analysis was performed using the Mann–Whitney U test, the Kruskal–Wallis test, and Spearman’s rank correlation coefficient. **Results:** A significantly higher expression level of *MMP13* mRNA was noticed in the OA-affected articular cartilage with subchondral bone compared to the control tissue (*p* = 0.027, Mann–Whitney U test). The expression level of *MMP13* mRNA was higher in patients with stage 4 knee OA than in those with stage 3, but the difference in *MMP13* mRNA expression level was statistically insignificant (*p* > 0.05, Mann–Whitney U test). A higher *MMP13* mRNA expression level was noticed in the OA-affected synovium compared to the control tissue (median RQ: 0.068 and 0.037, respectively), but these differences were not significant (*p* > 0.05, Mann–Whitney U test). A significantly higher *MMP13* mRNA expression level was observed in the synovium of stage 4 knee OA patients compared to stage 3 patients (*p* = 0.015, Mann–Whitney U test). There was no significant difference in the expression level of *MMP13* mRNA between both tissues, i.e., the articular cartilage with subchondral bone and the synovium from the stage 3 group and the control tissue (*p* > 0.05, Mann–Whitney U test); however, a significant difference was found between these tissues in stage 4 and in the control tissue (*p* = 0.014, Mann–Whitney U test). **Conclusions:** The results of our pilot study indicated the diagnostic potential of *MMP13* mRNA and proved its role in the development and progression of OA. Further studies are needed to verify the potential utility of *MMP13* mRNA in the development of molecularly targeted therapy for patients with OA.

## 1. Introduction

The most common progressive degenerative joint disease is osteoarthritis (OA), which is known to affect all joint tissues, including the articular cartilage, subchondral bone, ligaments, capsule, and synovium, meniscus, and intra-articular adipose tissues. The disease has a significant negative impact on patients’ lives and can lead to functional limitations and disability [1]. In nearly three decades, the incidence of OA cases has increased worldwide by 113.25% [2], and its prevalence is expected to reach 35% by 2030—a fact which has been attributed to the increase in obesity and to the number of elderly people in the population. In addition to aging and obesity, the development of OA is also exacerbated by traumatic knee injury, genetic predisposition, and abnormal mechanical stress [3]. It should be pointed out that gender, age, and BMI are recognized risk factors for the development of OA [4,5,6].

A significant role in the development and progression of OA is played by extracellular matrix metalloproteinases (MMPs), which are enzymes that degrade extracellular matrix (ECM) components [7,8,9]. Matrix metallopeptidase 13 (MMP-13) has a predominant role in OA because of its ability to degrade fibrillar molecules, including collagen types I, II, and III, and proteoglycans such as aggrecan [10]. Additionally, cartilage breakdown products stimulate the type A synoviocytes in synovium to release various inflammatory cytokines and MMPs, including MMP-13 [11,12]. It has been documented, in human and on animal models, that changes in the MMP-13 immunoexpression level may be associated with the hypertrophic phenotype of chondrocytes and the progression of cartilage damage [13,14]. Moreover, synovitis progression is associated with greater cartilage damage and OA progression, as defined by a poorer Kellgren and Lawrence (KL) grade or the narrowing of the joint space [15].

The symptoms of the disease include stiffness, swelling, decreased range of movement of the knee joint, and joint deformity, accompanied by pain [16]. Pain is often considered an important warning signal that plays a protective role in the response to acute tissue damage and inflammation; however, when acute pain persists and transitions into chronic pain, its control becomes much more challenging [17,18]. OA patients often experience pain that is initially activity-related and then becomes more constant over time, contributing to the development of a chronic disabling disease; this can have a negative impact on mental health, sleep, and social participation [19].

The pathogenesis of pain in osteoarthritis is not well understood. Although hyaline cartilage is not innervated, the subchondral bone or synovium contain nerve endings that may be a source of the nociceptive stimuli generated by tissue damage during joint degradation in OA [20]. Cartilage degradation is associated with greater pain perception and is linked to an imbalance between MMPs and tissue inhibitors of metalloproteinases (TIMPs) [21]. The innervated infrapatellar fat pad (IFP) may be responsible for anterior knee pain and may also participate in the development of knee osteoarthritis by secreting cytokines and adipokines into the surrounding tissues [22]. However, pain is a subjective and complex sensory experience, and its assessment is quite difficult [23]. One method for assessing pain is the visual analog scale (VAS), which is a routine clinical psychometric measurement tool designed to classify symptom severity and monitor the course of diseases such as OA [24,25]. As confirmed by Begum and Hossain’s research, VAS is a reliable and valid pain measurement scale [26].

Despite the studies of various authors confirming the involvement of MMP-13 in the pathogenesis of OA, to the best of our knowledge, there is not direct genetic evidence linking changes in the expression level of *MMP13* mRNA with the progression of OA, and no research has been conducted on a human model. A focus on this aspect of *MMP13* mRNA allowed for better monitoring of OA progression. Therefore, the topic seems to be particularly important from the point of view of developing a potential biomarker for the development and progression of OA in the knee. In the course of OA, early diagnosis and treatment play a key role in avoiding disability. There is still a need to search for early diagnostic markers related to the process of primary change that leads to damage in the articular cartilage. Following the need for such analysis, the aim of this study was not only to determine but to profile the expression of *MMP13* mRNA in the articular cartilage with subchondral bone and synovium from the affected and unaffected areas of the knee joint in patients with knee OA. To investigate whether this potentially dysregulated matrix metalloproteinase MMP-13 could also be associated with specific clinical features of the disease, we analyzed the level of *MMP13* mRNA expression in the collected biological material with regard to sex, age, body mass index (BMI), and also disease stage. Moreover, differences in the level of *MMP13* mRNA expression were compared with the degree of pain perception; the findings could indicate whether this molecular factor may serve as a biomarker related to pain in OA patients and whether it may be a prospective target for pain-relieving therapy.

The aim of the study was to identify the change in *MMP13* mRNA expression level in the articular cartilage with subchondral bone and synovium from the affected and unaffected areas of the knee joint in patients with knee OA. The study also analyzed the differences in the expression level of *MMP13* mRNA in the articular cartilage with subchondral bone and synovium of OA patients, with regard to sex, age, body mass index (BMI), and also disease stage, in order to develop a potential indicator of OA progression. Moreover, differences in the level of *MMP13* mRNA expression were compared with the degree of pain perception; the findings could indicate whether this molecular factor may be served as a biomarker related with pain in OA patients and a prospective target for pain-relieving therapy.

## 2. Materials and Methods

### 2.1. Clinical Characteristics of the Patients

**Figure 1:**
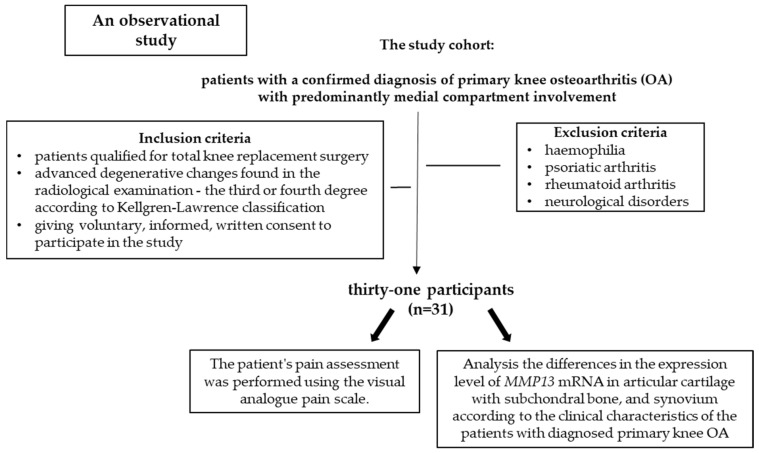
Flow diagram for participant selection.

The study cohort included 31 patients (n = 31) with a confirmed diagnosis of primary knee osteoarthritis (OA) with predominantly medial compartment involvement. The cross-sectional study involved 21 women and 10 men aged 54 to 75 (mean age 68 ± 7 years), with height ranging from 1.46 to 1.83 m (mean 1.64 m ± 0.08 m) and with weight ranging from 66 to 112 kg (average 88 kg ± 12.12 kg). Consecutive sampling was employed to recruit participants to the study. For inclusion in the study, the participants had to demonstrate advanced degenerative changes in a radiological examination: stage 3 or 4, according to the Kellgren–Lawrence classification, as well as exhibiting clinical symptoms of OA. The criterion for exclusion comprised a diagnosis of rheumatoid arthritis, hemophilia, psoriatic arthritis, and neurological disorders (Figure 1). All eligible patients gave their voluntary, informed, written consent to participate in the study.

Fourteen patients with stage 3 knee OA (45% of the study group) and 17 with stage 4 (55% of the study group) were qualified for the study. As part of the treatment, the patients underwent total knee arthroplasty at the Department of Orthopedics and Traumatology, University Clinical Hospital No. 2 of the Medical University of Lodz, Lodz, Poland during the years 2019–2022. The clinical features of the patients diagnosed with primary knee OA are demonstrated in Table 1.

### 2.2. Acquisition and Storage of Biological Samples

During surgery, fragments of articular cartilage with a layer of subchondral bone and fragments of the synovium (up to a volume of 1 cm^3^) were collected from the affected area (medial compartment of the knee joint) and the unaffected area (lateral compartment of the knee joint); the latter served as a control. The collected biological materials were stored in Falcon tubes containing RNAlater Solution RNA stabilizing buffer (Ambio, Applied Biosystems, Carlsbad, CA, USA) and kept at 4 °C during transportation to the molecular laboratory. Then, samples were removed from the stabilizing buffer, fragmented, and placed at −80 °C for 24 h.

### 2.3. RNA Isolation from Tissue, and Qualitative and Quantitative RNA Evaluation

The fragments of articular cartilage, together with the subchondral bone layer, were crushed using a laboratory mortar, following which, all fragments of tissue, i.e., the crushed articular cartilage together with the subchondral bone layer and fragments of the synovium, were homogenized using an IKA homogenizer (IKA Werke GmbH & Co. KG, Staufen, Germany). Total RNA was then isolated from tissue homogenates using the mirVana miRNA Isolation Kit (Life Technologies, Carlsbad, CA, USA), in accordance with the manufacturer’s protocol. Qualitative and quantitative evaluation of RNA was performed spectrophotometrically (260/280 nm) with the Eppendorf BioPhotometerTM Plus apparatus (Eppendorf, Hamburg, Germany).

### 2.4. Evaluation of Relative Expression Level (RQ) of Genes

Reverse transcription (RT) for genes was performed with the use of the High-Capacity cDNA Reverse Transcription Kit (Applied Biosystems, Carlsbad, CA, USA) in a Personal Thermocycler (Eppendorf, Germany), according to the manufacturer’s protocol.

The relative expression level of *MMP13* mRNA was analyzed in a 7900HT Fast Real-Time PCR System (Applied Biosystems, Carlsbad, CA, USA) using TaqMan assay: *MMP13* (Hs00942584_m1) and *HPRT1* (Hs02800695_m1). The latter served as an endogenous control. The relative expression of *MMP13* mRNA was determined using the delta–delta CT method (TaqMan Relative Quantification Assay software, Applied Biosystems). Reference total RNA from human chondrocytes—HC-a tRNA (ScienCell Research Laboratories, Carlsbad, CA, USA) and synovium (OriGene Technologies, Rockville, MD, USA) were used as calibrators; their expression was assumed to be RQ = 1.

### 2.5. Statistical Analysis

The RQ values for the studied genes are shown as median values. For all statistical analyses, the level of statistical significance was adopted at *p* < 0.05. The Shapiro–Wilk test was used to test the normality of data. As the distribution was not normal, non-parametric tests were conducted depending on the size of the groups: the Mann–Whitney U test was utilized for two-group comparisons and the Kruskal–Wallis test for multiple group comparisons. Spearman’s rank correlation coefficient was used to determine the direction and strength of the relationship for individual variables. The statistical analysis was performed using Statistica 13.1 software (StatSoft, Cracow, Poland).

## 3. Results

### 3.1. Relative Expression Level of the *MMP13* mRNA in OA-Affected Articular Cartilage with Subchondral Bone vs. Control Tissue

The expression level of *MMP13* mRNA was upregulated (RQ > 1) in all (n = 31) OA-affected articular cartilage with subchondral bone and in all (100%n = 31) control tissue fragments. A significantly higher expression level of *MMP13* mRNA was noticed in OA-affected articular cartilage with subchondral bone compared to the control tissue (median RQ: 89.061 and 31.025, respectively) (*p* = 0.027, Mann–Whitney U test).

### 3.2. Relative Expression Level of the *MMP13* mRNA in Articular Cartilage with Subchondral Bone of Patients with Stage 3 and 4 Knee OA vs. Control Tissue

**Figure 2:**
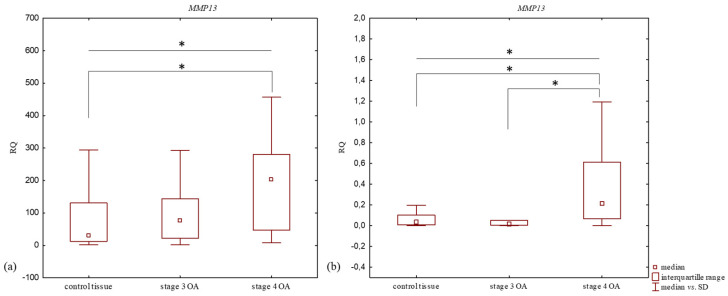
Box plot presenting differences in median RQ values for *MMP13* in (**a**) OA-affected articular cartilage with subchondral bone and (**b**) OA-affected synovium according to stage of disease vs. control tissue. * *p* < 0.05.

Significant differences were found between *MMP13* mRNA expression level in the articular cartilage with subchondral bone of patients in stage 3 of the disease, and those in stage 4, and with the control tissue (*p* = 0.0496, Kruskal–Willis test) (Table 2, Figure 2). Patients with stage 4 knee OA demonstrated a higher expression level of *MMP13* mRNA than the stage 3 OA group. No significant difference in *MMP13* mRNA expression level was noted between the articular cartilage with subchondral bone samples from stage 4 and stage 3 (*p* > 0.05, Mann–Whitney U test). An insignificant difference in *MMP13* mRNA expression level was noted between the articular cartilage with subchondral bone from the stage 3 group and the control tissue (*p* > 0.05, Mann–Whitney U test); however, a significant difference was observed between the articular cartilage with subchondral bone from the stage 4 and control tissue (*p* = 0.014, Mann–Whitney U test).

### 3.3. Relative Expression Level of *MMP13* mRNA in Articular Cartilage with Subchondral Bone According to Clinical Features of Knee OA Patients (Sex, Age, and BMI)

The female group demonstrated a lower expression level of *MMP13* mRNA in articular cartilage with subchondral bone than the male group (median RQ: 84.548 and 167.072, respectively), as did the patients aged >65 compared to those aged ≤65 (median RQ: 89.061 and 107.976, respectively). However, the differences were statistically insignificant (*p* > 0.05, Mann–Whitney U test) (Table 3).

Regarding the BMI, the highest expression level of *MMP13* mRNA in OA-affected articular cartilage with subchondral bone was observed in overweight patients; the level of expression was lower in patients with class I obesity and was the lowest in patients with class II + III obesity (Table 3). The differences in *MMP13* mRNA expression levels were found to be insignificant (*p* > 0.05, Kruskal–Willis test).

### 3.4. Relative Expression Level of *MMP13* mRNA in Articular Cartilage with Subchondral Bone of Knee OA Patients According to Experienced Pain Intensity

**Figure 3:**
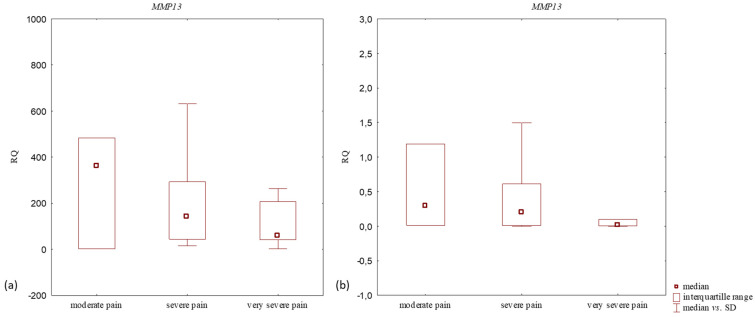
Box plot presenting differences in median RQ values for *MMP13* in (**a**) OA-affected articular cartilage with subchondral bone and (**b**) OA-affected synovium according to pain intensity (based on VAS scores).

The highest *MMP13* mRNA expression level in articular cartilage with subchondral bone was noted in patients with moderate pain, and the lowest expression in those with very severe pain (Table 3). No statistically significant differences in *MMP13* mRNA expression levels were found between the study groups (*p* > 0.05, Kruskal–Willis test) (Figure 3). *MMP13* mRNA expression level in articular cartilage with subchondral bone of patients with knee OA did not correlate with pain intensity (*p* > 0.05, rho = −0.05, and Spearman’s rank correlation).

### 3.5. Relative Expression Level of *MMP13* mRNA in OA-Affected Synovium vs. Control Tissue

*MMP13* mRNA expression level was downregulated (RQ < 1) in all (n = 31) OA-affected synovium samples and in 81% of control tissue fragments (n = 25). A higher *MMP13* mRNA expression level was noticed in the OA-affected synovium compared to the control tissue (median RQ: 0.068 and 0.037, respectively), but these differences were not significant (*p* > 0.05, Mann–Whitney U test).

### 3.6. Relative Expression Level of *MMP13* mRNA in Synovium of Knee OA Patients with Stage 3 and 4 vs. Control Tissue

Statistically significant differences were found between *MMP13* mRNA expression level in the synovium of knee OA patients in stages 3 and 4, and the control tissue (*p* = 0.005, Kruskal–Willis test) (Table 2, Figure 2). The control synovium demonstrated a higher *MMP13* mRNA expression level than the synovium of stage 3 knee OA patients, but the difference was statistically insignificant (*p* > 0.05, Mann–Whitney U test). A significantly higher *MMP13* mRNA expression level was observed in the synovium of stage 4 knee OA patients compared to the control tissue (*p* = 0.002, Mann–Whitney U test). Statistically significant higher *MMP13* mRNA expression levels were observed in the synovium of stage 4 knee OA patients compared to stage 3 patients (*p* = 0.015, Mann–Whitney U test).

### 3.7. Relative Expression Level of the *MMP13* mRNA in Synovium According to Clinical Features of Knee OA Patients (Sex, Age, and BMI)

Among the patients with knee OA, the female group demonstrated a lower *MMP13* mRNA expression level in the synovium than the male group (median RQ: 0.021 and 0.168, respectively); in addition, patients aged ≤65 presented higher levels than those aged >65 (median RQ: 0.307 and 1.125, respectively). However, the differences were not statistically significant (*p* > 0.05, Mann–Whitney U test).

With regard to BMI, patients with class II + III obesity demonstrated a higher *MMP13* mRNA expression level in the synovium than those with class I obesity; however, their level was lower than that in overweight patients (Table 3). The differences in *MMP13* mRNA expression level are statistically insignificant (*p* > 0.05, Kruskal–Willis test).

### 3.8. Relative Expression Level of *MMP13* mRNA in Synovium of Knee OA Patients According to Experienced Pain Intensity

The patients with moderate pain indicated the highest *MMP13* mRNA expression level in the synovium while those with very severe pain demonstrated the lowest levels (Table 3). The differences in *MMP13* mRNA expression levels between the study groups were statistically insignificant (*p* > 0.05, Kruskal–Willis test) (Figure 3). A statistically insignificant very weak negative correlation was found between *MMP13* mRNA expression level in the synovium of knee OA patients and pain intensity (*p* > 0.05, rho = −0.14, and Spearman’s rank correlation).

## 4. Discussion

Osteoarthritis is a very common degenerative joint disease characterized by progressive loss of joint cartilage and changes in the subchondral bone, meniscal degeneration, osteophyte formation, and inflammation and fibrosis of the synovial membrane and infrapatellar fat tissue [4,28]. Globally, around 86.7 million people are believed to have suffered from knee osteoarthritis in 2020 [4], with a global incidence of 203 per 10,000 person-years. Although it is known that predisposition, aging, obesity, mechanical stress, and traumatic joint injury play roles in OA, the exact etiology of this disease remains largely unknown. Progression eventually leads to joint disability due to the poor repair capacity of cartilage [28]. A complete understanding of the mechanisms underlying OA progression might enable the development of more efficient methods of treatment. Unfortunately, no effective therapy that stops the structural degradation of cartilage and bone or reverses existing structural defects currently exists [11,29].

Despite numerous comprehensive studies focused on discovering a molecular biomarker of OA at the gene, protein, and metabolite level in different types of samples and joints affected by this disease, none have been validated as sensitive and specific for the diagnosis of patients suffering from OA [30]. So far, the diagnosis of OA is mainly made clinically, often being confirmed by radiography [31]. The search for genomic biomarkers of OA has been a research priority for decades, as their identification would enable population-scale screening and early detection, reducing the burden of OA. Many studies focusing on gene expression assessment were excluded because they did not adequately account for important covariates such as age and gender [30]. Therefore, much progress is still needed to understand OA’s heterogeneity, develop clinically useful genetic biomarkers of OA, and improve disease management. Recently, proteomic analysis has been used to discover OA biomarkers, and this is a powerful tool in the global assessment of protein expression that has been widely applied to biomarker discovery in clinical diseases [32,33,34]. Proteomic analyses of the SF from patients with OA and controls revealed significant change in expression levels of α-enolase (ENO1) and fibrinogen β-chain, suggesting the diagnostic potential of these proteins [32]. Proteomic analysis by Paz-Gonzalez et al., comparing healthy and diseased menisci from mildly degenerated and end-stage OA joints, identified 42 candidate protein biomarkers, and additional qualitative analysis demonstrated that lower levels of cytokine-like protein 1 (CYTL1) may serve as a potential biomarker of early joint degradation [33]. Proteomics analysis of knee subchondral bone by Tan et al. differentially identified proteins’ expression profiles associated with OA, and further biological information analysis showed that a majority of these proteins participated in the dysregulation of the complement and coagulation cascades [34]. They revealed that the expression level of protein C inhibitor (PCI, also known as SerpinA5) was significantly increased in the severely damaged subchondral bone of patients with OA compared to the control group and suggested change in the expression level of this protein could be used as a potential OA biomarker candidate. Of note, OA is considered a systemic disease that affects the circulatory system, which makes serum and plasma analysis crucial to advancing our understanding of OA as it can provide information on the mechanisms of the disease, its progression, and response to treatment [30]. Mehta et al. performed serum metabolome analysis to discover biochemical alteration in symptomatic knee OA patients, which also can be associated with knee pain [35]. They showed the levels of certain proinflammatory cytokines such as interleukin 1β (IL-1β), interleukin 2 (IL-2), and tumor necrosis factor α (TNF-α) correlated negatively with pain, while levels of interferon-gamma (IFN-γ) had a positive correlation. It is known that OA patients often experience acute and chronic inflammation involving multiple tissues within the joint, promoting its structural degradation, as well as outside the joint (systemic inflammation), which may contribute to the diverse perception of pain and symptoms in these patients [36].

A disconnect has been observed between clinical patient symptoms in OA and radiographic findings, which may affect inflammation [36,37]. The recent scientific literature has described that abnormalities in the gut microbiome (so-called dysbiosis) can lead to systemic inflammation, especially in the case of an impaired intestinal barrier, and can contribute to the exacerbation of chronic pain through the production of neurotransmitters and affect the perception of pain via the gut–brain axis [38]. Moreover, in vivo studies showed that nutrients in the diet and prebiotics can improve the status of OA by modulating the microenvironment of the gut microbiota, which leads to a reduction in joint damage, senescent cells, leptin levels, lipids, endotoxins, and inflammatory factors [39]. In vivo study conducted by Cho et al. revealed after injected live Lactobacillus once daily for 3 weeks into rats with monosodium iodoacetate (MIA)-induced OA (male Wistar rats) decreased the expression of transient receptor potential cation channel subfamily V member 1 (TRPV1) and calcitonin gene-related peptide (CGRP) in the dorsal root ganglion (DRG), resulting in a reduction in pain [40]. Additionally, cartilage destruction was markedly decreased in the LA-1-treated group compared to the control group, which is also consistent with the decreased levels of the inflammatory markers: interleukin 1β (IL-1β), inducible nitric oxide synthase (iNOS), monocyte chemoattractant protein 1(MCP-1), TNF-α, and also MMP-13. The results of a study by Gonzalez-Alvarez showed lifestyle changes, such as a diet rich in probiotic foods and exercise, may reduce pain experience and improve the quality of life for OA patients through beneficial changes in the composition of the gut microbiome [38]. Physical activity not only directly affects the central nervous system (CNS), modifying pain perception and cognitive processing, but also positively influences the gut microbiome via increases diversity and the amount of beneficial taxa and metabolites, thus, it may serve as a potential preventive and therapeutic measure [38]. The way to relive pain in OA patients with chronic musculoskeletal pain may be to use orthopedic manual therapy (OMT) techniques, in which a mechanical stimulus initiates a cascade of peripheral and central neurophysiological effects, decreasing proinflammatory mediators, and activates supraspinal inhibitory pathways to produce a hypoalgesic and antinociceptive response [41,42]. However, as the umbrella review by Martínez-Pozas et al. showed, OMT consisting of passive or active joint mobilization, joint manipulation, or neurodynamic techniques performed in isolation on patients with chronic musculoskeletal pain improved pain sensitization, but its efficacy was limited to immediate but short-term effects [42]. This is why it is so important to use a multidimensional treatment program for patients with OA to relieve pain.

Articular cartilage is the connective tissue covering the ends of bones in joints. It facilitates the transfer of loads to the joint surfaces, thus allowing smooth and friction-free movement. Chondrocytes synthesize ECM and directly interact with it to form the unique structure of articular cartilage that allows it to perform its function. The ECM is composed of two main components, viz. collagens and proteoglycans, and provides a suitable microenvironment for chondrocytes. During the development of OA, the molecular composition and organization of the ECM in the articular cartilage changes due to a shift in the metabolic activity of chondrocytes, which produce more catabolic factors involved in cartilage degradation; these include ECM-degrading enzymes such as MMPs, a disintegrin and metalloproteinase with thrombospondin motifs (ADAMTSs), which break down proteoglycans and the collagen network, disrupting the integrity of the cartilage [7,8]. Consequently, changes occur in the OA cartilage’s biomechanical behavior: there is a reduction in the tensile strength of the ECM and the shear stresses produced by friction between the adjacent bones of the articulation increase [43].

MMPs are a family consisting of at least 28 structurally related, zinc-dependent proteolytic enzymes that can degrade all components of the extracellular matrix, including collagen, vitronectin, fibronectin, laminin, and proteoglycans [7]. When chondrocytes and synoviocytes are subjected to severe mechanical stress and trauma in the joints, the release of proinflammatory cytokines such as IL-1β, TNF-α, and TGF-β promotes the expression of MMPs. Most MMPs, including MMP-1, MMP-2, MMP-3, MMP-8, MMP-9, MMP-10, MMP-13, MMP-14, and MMP-16 are associated with a change in the ECM architecture and with articular cartilage degradation in OA [7,44]. There have been some studies indicating the potential role of MMPs as biomarkers for OA, however, theirs authors focused on the assessment of protein profiles [8,44,45,46,47]. A study by Pengas et al. revealed that the MMP-3 immunoexpression level was upregulated in the synovium of OA patients, and positively correlated with radiographic scores, as well as with its immunoexpression level in serum [45]. They suggested a change in the serum immunoexpression level of MMP-3 may be used as a potential and easily measurable biomarker for knee OA, using a simple blood test. In turn, Singh et al. assessed the ability of serum MMP-3 immunoexpression levels to differentiate between a normal knee and a knee with primary knee OA and its ability to differentiate between various severity grades [46]. They revealed a significantly increased serum MMP-3 immunoexpression level in OA patients compared to the control group, however its immunoexpression level had moderate positive correlation with OA progression when assessing according to the Kellgren–Lawrence classification. Slovacek et al. assessed whether serum MMP-9 and ADAMTS4 immunoexpression levels could be helpful in diagnosing patients with OA [44]. The results of their study revealed a significantly increased level of MMP-9 immunoexpression in patients with OA compared to the control group, but the level of ADAMTS-4 immunoexpression did not show significant differences between the study groups. Meehan conducted a study to assess changes in the MMP and cytokine profiles in the synovial fluid of OA patients, which revealed an increase in the immunoexpression level of MMP-1,IL-2, IL-6, and TNF-α, but decreases in the immunoexpression level of MMP-2, MMP-3, MMP-8, and IL-4 with OA development [47]. In patients with OA, the immunoexpression levels of TNF-α, IL-1β, IL-7, MMP-1, MMP-2, and MMP-3 were significantly higher in patients with active rheumatoid arthritis (RA), whereas the immunoexpression levels of IL-1, IL-2, IL-4, IL-6, IL-15, IL-18, and MMP-3 were not statistically different. The authors’ research indicates that OA patients have a proinflammatory environment similar to RA patients, which may contribute to ECM degradation and progressive cartilage loss. A recent study by Xin et al. showed that assessment of serum MMP-13 immunoexpression levels may be valuable in diagnosing, measuring disease severity, and predicting OA, but only in patients with advanced stages of the disease [8]. Therefore, in our research, we decided to investigate in more detail what changes in the *MMP13* expression at the transcriptional level occur within various tissues of the knee joint during OA development and progression of the disease and whether these changes correspond to the pain sensations of patients. In our opinion, further studies are needed to identify biomarkers of cartilage degradation and inflammation, which could indicate an early stage of OA development, as this is a promising area of research to understand the pathogenesis of this disease.

MMP-13 is involved in the degradation of matrix molecules such as collagen types I, II, III, IV, IX, X, perlecan, osteonectin, and proteoglycan; however, its preferred substrate is type II collagen, which is cleaved five times faster than type I collagen and six times faster than type III collagen. It also works faster than other collagenases [11]. MMP-13 expression and its activity are strictly regulated by a number of molecular and epigenetic mechanisms [11,48], but are disturbed during the development and progression of OA [11,49,50].

Our study indicated a significantly higher *MMP13* mRNA expression level in OA-affected articular cartilage with subchondral bone compared to the control tissue. This findings is consistent with Reboul et al., who report increased expression of MMP-13 at mRNA and protein levels in cartilage in OA patients [51]. Moreover, our study revealed *MMP13* mRNA expression was elevated in articular cartilage with subchondral bone during OA progression. Our findings are the first data regarding the level of *MMP13* mRNA expression according to OA progression in human articular cartilage with subchondral bone. An in vivo study by Wang et al. found cartilage-specific deletion of the *MMP13* gene or inhibition of MMP-13 activity decelerated the progression of OA in a murine model of injury-induced knee OA, which could be used as a potential therapeutic strategy for the prevention and treatment of OA [49]. MMP-13 plays an important role in regulating the viability of chondrocytes, and deletion of the *MMP13* gene significantly reduces the apoptosis of chondrocytes [49]. In addition, an in vivo study by Kamekura et al. showed MMP-13 expression has a similar localization to type X collagen in cartilage, which is known a marker of chondrocyte *hypertrophy.* The authors suggest that MMP-13 can be produced by pathological hypertrophic chondrocytes even in the early stage of OA development [14]. As revealed in a study by Wei et al., *MMP13* and *COLX* mRNA expression levels correlate with changes in chondrocyte morphology, hypertrophy, and severity of cartilage damage, and this was assessed using the modified Mankin grading system [13].

More and more evidence indicates that inflammation of the synovial membrane also plays an important role in the progression of OA [15]. As demonstrated by Hu and Ecker, cartilage breakdown products are released into the synovial fluid and phagocytosed by resident macrophages, such as type A synoviocytes, but, when the production of these decaying particles exceeds the system’s ability to eliminate them, type A synoviocytes adopt an inflammatory profile, producing various inflammatory cytokines like TNF-α, IL-1, and IL-6, and MMPs such as MMP-13. These, in turn, favor the catabolic effect on chondrocyte metabolism, accelerating the progression of OA [11].

Our study revealed a higher *MMP13* mRNA expression level in the OA-affected synovium than in the control tissue; however, insignificant differences were noted between the two. Significant differences were noted in the *MMP13* mRNA expression level in the synovium according to OA progression (*p* = 0.015, Mann–Whitney U test). A higher expression level of this gene was found in group of patients with stage 4 than in those with stage 3. These findings suggest that *MMP13* mRNA expression level in the synovium may be a potential biomarker for OA progression. Jarecki et al. report a significantly higher expression level of pro-*MMP13* protein in the synovial fluid of patients with more advanced stages according to the Kellgren–Lawrence classification [52]. Özler et al. also noted a higher MMP-13 protein level in the synovial fluid in stage 4 OA compared to stage 3 [53]. Hence, MMP-13 appears to play a significant part in OA progression. An increased *MMP13* mRNA expression level in OA-affected synovial membrane may be caused by the transition of type A synoviocytes to the inflammatory state and a progressive development of inflammation in knee joints [11,15].

OA is a complex, heterogeneous condition and is multifactorial in origin [54]. It is approximately 1.7-times more common in women than men [52]. Women not only suffer from OA more often than men, but also have a more severe course [55]. The anatomy of the knee joint, cartilage degeneration and regeneration, and the effects of sex hormones on cartilage differ between the sexes; for example, women have thinner articular cartilage and lower knee cartilage volume than men [5]. Women also have a greater tendency to various joint instability, and uneven mechanical load, which can influence susceptibility. They also present with a higher normalized contact area and with lower congruity index values than men, and, thus, have a higher risk of OA development [56]. They also experience more severe cases of knee arthritis than men and are more likely to be candidates for total knee replacement surgery [5,57].

We also examined whether *MMP13* mRNA expression levels vary according to sex. Our findings did not reveal any significant differences between men and women with regard to *MMP13* mRNA expression in the articular cartilage with subchondral bone or synovium. Similar results were obtained by Aref-Eshghi et al. [58].

The risk of developing cartilage degeneration increases with age [5]. It is estimated that 10% to 15% of all adults over the age of 60 have some degree of OA [59]. Primary knee OA mainly occurs in older people as a result of wear and tear of the cartilage tissue, while younger people may develop secondary knee OA as a result of joint overload or trauma. The incidence of OA increases dramatically in women, mainly after the age of 50, around the time of menopause. Several studies have shown that a decline in sex hormone levels, particularly estrogen deficiency, during menopause was associated with an increased risk and incidence of OA [60,61]. Jin et al report that lower serum estradiol, testosterone, and progesterone were associated with increased knee effusion-synovitis in post-menopausal women [62]. Our study group also reflects the global trend in disease prevalence: women constituted 68% of the group, of whom most were older patients with OA, i.e., aged 56–75.

Our analysis found *MMP13* mRNA expression levels in OA-affected articular cartilage with subchondral bone and synovium to be lower in patients aged >65 than in those aged ≤65; however, these differences were statistically insignificant. Rai et al. found a negative correlation between age and the expression of *MMP13* mRNA in the meniscus [6]. Brophy et al. found that the change in *MMP13* mRNA expression level in older individuals is the result of age-related changes in the expression levels of proinflammatory cytokine genes, such as *IL1B*, *IL6*, and *TNF*, which may be downregulated [63].

Obesity is considered to be one of the major risk factors for both the occurrence and progression of OA, and this applies to both weight-bearing and non-weight-bearing joints. It is well known that abnormal mechanical loading caused by increased body weight loading the joints is responsible for the initiation and progression of obesity-induced OA [64]. Weight overloading of joints has been shown to upregulate the secretion of TNFα and IL-1, thus mediating the degradation of cartilage ECM via MMPs, including MMP-13 [65,66,67,68]. Nevertheless, inflamed adipose tissue and dyslipidemia are believed to play pivotal roles in obesity-induced OA [68]. Various adipokines such as leptin, adiponectin, and resistin influence the pathogenesis of OA via regulation of MMP-13 production in human osteoarthritic cartilage [69,70,71].

No significant differences in *MMP13* mRNA expression levels were noted in the articular cartilage with subchondral bone and synovium in patients with OA who were overweight or who suffered from class I obesity and class II + III obesity. However, the *MMP13* mRNA expression level in cartilage was found to be negatively related to the degree of obesity. Interestingly, Rai et al. found the expression levels of *MMP1*, *MMP3*, and *MMP9* in articular cartilage to negatively correlate with BMI, but this was not the case for *MMP13* [6]. Further analyses using rank-order partial correlation of these genes’ expression level with age, controlling for BMI, found age to have a greater impact on the gene expression pattern in articular cartilage than BMI [6]. In the present study, the apparent negative relation between *MMP13* mRNA expression levels and BMI can be accounted for by age differences.

Symptoms of OA include stiffness, swelling, decreased range of movement of the knee joint, and joint deformity. People with symptomatic knee OA may suffer from either intermittent or constant knee pain. Usually, the early stages of OA are characterized by intermittent, intense pain triggered by some activity, while in the advanced stages of this disease, constant pain is often present, especially at night [72]. Persistent pain usually leads to physical disability, and difficulty in conducting activities of daily living (ADL) [72,73,74]. Pain related to OA is a major predictor of reduced physical activity in the elderly, and this is exacerbated by the fact that physical inactivity also leads to decreased muscle strength [73,75].

Patients with OA have been shown to respond differently to various analgesics, suggesting that there may be many distinct pain phenotypes associated with the disease [76]. The source of pain in patients with OA is not particularly well understood. The experience of pain may be caused by a variety of factors, including joint degeneration, and extra-articular sources of pain such as neural and psychosocial factors [77]. Osteoarthritis in women appears to be more symptomatic due to a different response to inflammatory stimuli. Different molecular mechanisms, signaling pathways, and expression of inflammatory cytokines have been revealed between sexes, which may explain the variable pain perception in arthritis-induced pain [78]. Moreover published data suggest that woman, mostly during perimenopause and menopause, experience stronger and more persistent pain compared to men, due to hormonal disorders, including a decrease in estrogen levels, which has a significant impact on the proper functioning of muscles and joints [79]. Of course, the causes of pain during OA could be multifactorial, where, in addition to hormonal factors, genetic, anatomic, and different earlier injuries also play an important role [80,81].

Studies have been conducted to target pain based on its mechanisms in patients with OA, but none have been approved to date [82]. The current pain management strategies for OA cannot provide satisfactory pain relief, and chronic use of commonly-administered analgesics, such as NSAIDs, acetaminophen, and opioid painkillers, is often associated with significant side effects and toxicity [19]. Pain is the dominant factor influencing clinical interventions in OA patients and is the major reason for total joint arthroplasty [83]. There is a need to identify the molecular mechanisms contributing to the onset and maintenance of pain associated with knee OA, as this will play a key role in developing targeted and more effective pain treatments.

Traditionally pain is categorized into nociceptive pain, resulting from tissue damage, and neuropathic pain, involving nerve damage [84]. Increasing numbers of researchers suggest that pain in OA is characterized by a nociceptive and neuropathic component, as well as possible sensations that may be both mechanical (peripheral) and central (secondary hyperalgesia) [85,86,87]. One study found MMPs to be strongly involved in the generation, amplification, and maintenance of pain in various clinical conditions, including OA [21]. MMP-2 and MMP-9 are known to have roles in the induction of neuropathic pain and the inhibition of these MMPs may provide a novel therapeutic approach [88]. Moreover, a functional interaction has been identified between MMPs and the endocannabinoid system (ECS), which is involved in modulation of nociceptive processing during OA [89,90]. An in vivo study on male Wistar rats with induced knee OA found that the expression of selected MMPs (MMP-3, MMP-9, and MMP-13) in OA-affected cartilage corresponded to the two-stage development of OA pain and may have a role in the biphasic progression of OA-related pain [89]. Furthermore, the authors noted an increase in *MMP3*, *MMP9*, and *MMP13* expression levels over a time period that reflects the initiation of the chronic phase of OA-related pain (i.e., 14 days after induction of knee OA).

So far, no work has explored the association between *MMP13* mRNA expression level and the degree of pain in patients with knee OA. Hence, the present study examined the differences in *MMP13* mRNA expression level in articular cartilage with subchondral bone and synovium of patients with knee OA and pain perception measured by VAS. Our analysis found that the *MMP13* mRNA expression level in both OA-affected articular cartilage with subchondral bone and synovium decreases with higher degrees of pain perception among patients with knee OA; however, the differences in *MMP13* mRNA expression between the study groups were found to be statistically insignificant. Moreover, the *MMP13* mRNA expression level in the articular cartilage was not correlated with pain intensity, and only a very weak, statistically insignificant, negative correlation was found between the expression level of *MMP13* mRNA in synovium and pain perception. Li et al. showed a weak positive correlation between the immunoexpression level of MMP-13 in synovial fluid and the degree of pain sensation measured by a numeric rating scale (NRS) in patients with knee OA, but no correlations were found between this protein and pain perception measured by VAS [91]. In turn, a study by Olivotto et al. revealed significant differences in how pre-operative pain was observed in patients with positive MMP-13 immunoexpression in the meniscus compared to a negative group of patients; however, no impact of MMP-13 expression on post-operative clinical outcomes was observed after the 2-year follow-up [92].

As for the strengths of our work, we demonstrated a differential expression profile of the *MMP13* gene between paired OA-affected articular cartilage with subchondral bone and synovial membrane compared with control tissue from the same patient. Moreover, our findings are the first data on the expression level of *MMP13* mRNA in relation to the progression of OA in human articular cartilage with subchondral bone, which provides a basis for the conclusion that *MMP13* mRNA may be a biomarker of the progression of knee osteoarthritis (OA) and may serve in future to develop a potential therapeutic strategy for the prevention and treatment of this disease.

We encountered some inevitable limitations in this study. One of them is the fact that, according to the recommendations for total knee arthroplasty, biological material could only be collected from patients in advanced stages of OA, which influenced the size of the sample included in the study. Also, the studied OA patients only demonstrated slight variations in the degree of disease development and pain perception, which may be the main reason for the statistically insignificant correlations between the level of *MMP13* mRNA expression and the degree of pain. Also, a lack of information about the occupations and sports activities of our patients impoverished the discussion. A significant limitation of our study was the small sample size and the lack of the possibility to monitor confounding factors such as age, gender, and BMI, which results from this being a pilot, single-center study and our conducting the study at one time point. Due to the difficult in separating articular cartilage from adjacent subchondral bone tissue at the time of surgery, we, therefore, analyzed our material as a complex. The articular cartilage fragments with a subchondral bone layer and the synovial membrane fragments used in the study were collected from the same patients. Moreover, our study focused solely on the assessment of the *MMP13* mRNA expression level, so protein expression was not assessed, and inflammatory factors were not studied, although these could also be helpful in better understanding the mechanisms of disease progression.

## 5. Conclusions

A higher *MMP13* mRNA expression level was observed in OA-affected articular cartilage with subchondral bone compared to control tissue, which may be related to increased chondrocyte apoptosis and hypertrophic transformation of chondrocytes. An elevated *MMP13* mRNA expression level was also found in OA-affected synovial membrane compared to control tissue, which may be caused by the transition of type A synoviocytes to the inflammatory state and the development of inflammation. *MMP13* mRNA expression in both OA-affected articular cartilage with subchondral bone and synovium increased with the advancement of the disease. Changes in the *MMP13* mRNA expression level in articular cartilage with subchondral bone and synovium from knee OA patients may serve as potential molecular biomarkers of OA development and progression. Due to the results showing that the degree of pain intensity did not correlate with the level of *MMP13* mRNA expression in articular cartilage with subchondral bone and showed a very weak negative correlation with the level of expression of this gene in the synovial membrane, the assessment of this molecular factor cannot be used as a biomarker related to pain. However, there is a need to verify our observations in a larger group of patients, taking into account the mechanisms of controlling confounding factors.

## Figures and Tables

**Table 1:** The clinical characteristics of the patients with diagnosed primary knee OA.

Patient Characteristics	Number of Patients (n)	Total Percentage of Patients
Sex		
Female	21	68%
Male	10	32%
Age		
≤65 years	14	45%
>65 years	17	55%
Height		
≤1.64 m	17	55%
>1.64 m	14	45%
Weight		
≤88 kg	16	52%
>88 kg	15	48%
BMI ^a^		
25.0–29.9 (overweight)	4	13%
30.0–34.9 (class I obesity)	20	65%
35.0–39.0 (class II obesity)	6	19%
≥40 (class III obesity)	1	3%
Pain intensity according to VAS scores		
1–2 points (mild pain)	1	3%
3–4 points (moderate pain)	3	10%
5–6 points (severe pain)	15	48%
7–8 points (very severe pain)	11	36%
9–10 points (worst pain)	1	3%

^a^ BMI, calculated by dividing the body weight in kilograms by the height in meters squared [27].

**Table 2:** Median expression level (RQ value) of *MMP13* mRNA in OA-affected articular cartilage with subchondral bone and synovium according to stage of disease vs. control tissue.

	Expression Level of *MMP13* in Articular Cartilage with Subchondral Bone				Expression Level of *MMP13* in Synovium			
	Median RQ	IQR			Median RQ	IQR		
Control tissue (n = 31)	31.025	12.416—130.810		*p* = 0.0496	0.037	0.006—0.103		*p* = 0.005
Stage 3 of knee OA (n = 14)	76.508	21.635—143.728	*p* > 0.05		0.014	0.005—0.053	*p* = 0.015	
Stage 4 of knee OA (n = 17)	202.739	47.014—280.049			0.211	0.068—0.610		

IQR, interquartile range.

**Table 3:** Median expression level (RQ value) of *MMP13* mRNA in OA-affected articular cartilage with subchondral bone and synovium according to clinical characteristics.

Patient Characteristics	Expression Level of *MMP13* in OA-Affected Articular Cartilage with Subchondral Bone			Expression Level of *MMP13* in OA-Affected Synovium		
	Median RQ	IQR		Median RQ	IQR	
Sex						
Female (n = 21)	84.548	44.206—280.049	*p* > 0.05	0.021	0.011–0.586	*p* > 0.05
Male (n = 10)	167.072	37.279–208.109		0.168	0.053–0.404	
Age						
≤65 years (n = 14)	107.976	22.054–202.739	*p* > 0.05	0.307	0.006–0.195	*p* > 0.05
>65 years (n = 17)	89.061	57.574–364.510		0.125	0.014–1.144	
BMI						
overweight (n = 4)	297.213	97.529–544.589	*p* > 0.05	0.754	0.009–39.628	*p* > 0.05
class I obesity (20)	110.233	29.457–271.588		0.037	0.006–0.308	
class II + III obesity (n = 7)	74.85	44.206–202.739		0.284	0.034–5.978	
Pain intensity						
moderate pain (n = 3)	364.51	1.564–483.433	*p* > 0.05	0.284	0.013–1.192	*p* > 0.05
severe pain (n = 15)	143.728	44.206–292.745		0.195	0.011–0.610	
very severe pain (n = 11)	61.254	41.91–206.688		0.014	0.006–0.098	

## Data Availability

The data used to support the findings of this study are available from the corresponding author upon request.
